# Preparing for Mpox Resurgence: Surveillance Lessons From Outbreaks in Toronto, Canada

**DOI:** 10.1093/infdis/jiad533

**Published:** 2023-11-30

**Authors:** Herveen Sachdeva, Rita Shahin, Sylvia Ota, Sandra Isabel, Chand S Mangat, Rebecca Stuart, Shovita Padhi, Allison Chris, Sharmistha Mishra, Darrell H S Tan, Tom W Braukmann, AliReza Eshaghi, Edgard M Mejia, Nikho A Hizon, Michael Finkelstein

**Affiliations:** Toronto Public Health; Dalla Lana School of Public Health, University of Toronto; Toronto Public Health; Dalla Lana School of Public Health, University of Toronto; Toronto Public Health; Public Health Ontario, Toronto; Axe Maladies infectieuses et immunitaires, Centre de recherche du CHU de Québec–Université Laval; Wastewater Surveillance Unit, National Microbiology Laboratory, Public Health Agency of Canada, Winnipeg, Manitoba; Toronto Public Health; Toronto Public Health; Dalla Lana School of Public Health, University of Toronto; Toronto Public Health; Dalla Lana School of Public Health, University of Toronto; MAP Centre for Urban Health Solutions, Li Ka Shing Knowledge Institute, Unity Health Toronto; Department of Medicine, Temerty Faculty of Medicine; Institute of Health Policy, Management and Evaluation and Institute of Medical Science, University of Toronto; Institute for Clinical Evaluative Sciences (ICES); Division of Infectious Diseases, St Michael's Hospital, Toronto, Ontario, Canada; MAP Centre for Urban Health Solutions, Li Ka Shing Knowledge Institute, Unity Health Toronto; Department of Medicine, Temerty Faculty of Medicine; Institute of Health Policy, Management and Evaluation and Institute of Medical Science, University of Toronto; Division of Infectious Diseases, St Michael's Hospital, Toronto, Ontario, Canada; Public Health Ontario, Toronto; Public Health Ontario, Toronto; Wastewater Surveillance Unit, National Microbiology Laboratory, Public Health Agency of Canada, Winnipeg, Manitoba; Wastewater Surveillance Unit, National Microbiology Laboratory, Public Health Agency of Canada, Winnipeg, Manitoba; Toronto Public Health; Dalla Lana School of Public Health, University of Toronto

**Keywords:** mpox, genomics, wastewater, surveillance, outbreak

## Abstract

**Background:**

With many global jurisdictions, Toronto, Canada, experienced an mpox outbreak in spring/summer 2022. Cases declined following implementation of a large vaccination campaign. A surge in early 2023 led to speculation that asymptomatic and/or undetected local transmission was occurring in the city.

**Methods:**

Mpox cases and positive laboratory results are reported to Toronto Public Health. Epidemic curves and descriptive risk factor summaries for the 2022 and 2023 outbreaks were generated. First- and second-dose vaccination was monitored. Mpox virus wastewater surveillance and whole genome sequencing were conducted to generate hypotheses about the source of the 2023 resurgence.

**Results:**

An overall 515 cases were reported in spring/summer 2022 and 17 in the 2022–2023 resurgence. Wastewater data correlated with the timing of cases. Whole genome sequencing showed that 2022–2023 cases were distinct from 2022 cases and closer to sequences from another country, suggesting a new importation as a source. At the start of the resurgence, approximately 16% of first-dose vaccine recipients had completed their second dose.

**Conclusions:**

This investigation demonstrates the importance of ongoing surveillance and preparedness for mpox outbreaks. Undetected local transmission was not a likely source of the 2022–2023 resurgence. Ongoing preexposure vaccine promotion remains important to mitigate disease burden.

In spring 2022, mpox cases were reported globally in countries that were not previously endemic for the mpox virus (MPXV), including Canada [1]. Toronto is Canada's largest city. The first case in Toronto was a probable case with onset of symptoms on 28 April 2022. Toronto went on to experience Canada's largest outbreak, which predominantly affected gay, bisexual, and other men who have sex with men (gbMSM).

Public health interventions were put into place, including case and contact management [2]. Relevant incoming laboratory and physician reports were assessed to determine if they met the mpox case definition (confirmed, probable, or suspect) [3]. Case patients were interviewed and counseled about self-isolation. Backward contact tracing to determine the sources of infection and identification of exposures during the period of communicability were undertaken. Contacts were notified of exposures and advised to monitor for signs and symptoms of infection and receive postexposure vaccination if applicable. Community preexposure vaccine clinics started using the Modified Vaccinia Ankara-Bavarian Nordic vaccine on 12 June 2022. Eligible recipients at highest risk of exposure could get 1 dose of vaccine as part of an initial outbreak control strategy [4] as directed by the Ontario Ministry of Health. Limited eligibility for second doses started 24 August and was expanded on 30 September to all those eligible for preexposure prophylaxis. As in other previously nonendemic areas, we utilized community and health sector engagement, a communications strategy, enhanced surveillance, and preexposure vaccination (once available) to manage the response [5].

The number of mpox investigations and confirmed cases peaked in mid-July 2022 and declined thereafter. Case activity was sporadic by October 2022, indicating that the outbreak was coming under control. By late fall 2022, no new clinical cases were reported to Toronto Public Health (TPH). The outbreak was soon declared over, and domestic transmission was considered interrupted. However, after 6 weeks of no new case reports, cases appeared again in late December 2022, and a surge of new case reports occurred in early 2023. During this time, MPXV testing volume in Toronto declined, and emerging research reporting asymptomatic infections led to speculation that asymptomatic and/or undetected local transmission had been occurring locally.

Our traditional infectious disease system of clinical surveillance [6] and reporting of individual cases may not be reliable if asymptomatic and/or undetected transmission is occurring, and so we looked to nontraditional surveillance data sources to examine this question. The purpose of this study was to conduct a comparative epidemic appraisal of the 2022 outbreak and 2022–2023 resurgence by combining traditional clinical surveillance with whole genome sequencing (WGS) and wastewater-based surveillance (WBS) to explore potential sources of the resurgence. These results were used to inform strategies for outbreak control to mitigate the impact of the resurgence, as well as to better understand the utility of these complementary systems for surveillance of a novel communicable disease in our community.

## METHODS

We conducted a retrospective population-based analysis of all laboratory-confirmed and suspected mpox cases in Toronto using case surveillance, vaccination administration, wastewater surveillance, and viral WGS data.

### Case Surveillance

Health legislation in the province of Ontario, Canada, requires that suspect and/or confirmed mpox cases be reported to the local medical officer of health. All case data were entered into a provincial database and downloaded for analysis. Reports were received from treating physicians and laboratories and then investigated by public health officials to provide education and conduct contact follow-up, as well as to ascertain likely sources of acquisition, risk factors, symptoms, and severity of illness.

An epidemic curve was generated per weekly aggregated data for confirmed and probable cases up to 19 June 2023 by the best estimate of the date of disease onset—based on the earliest of symptom onset date, specimen collection date, or date that the case was reported to public health officials.

### Vaccination

Vaccine data were entered into and extracted from a provincial database (Panorama). Data are displayed by week of dose administration for those available up to 5 July 2023 and include all residents who received mpox vaccine in Toronto. Proportion of fully vaccinated individuals out of those initiating vaccination is calculated as the number of Toronto residents who received ≥2 doses, out of all receiving at least 1 dose.

### Wastewater-Based Surveillance

WBS consists of assessing the concentration of a pathogen of interest in human-source wastewater to estimate its associated infection prevalence in a community. In contrast to clinical surveillance, WBS is not limited in similar ways to clinical test-based underdiagnosis, provided that most infected individuals shed viral particles into wastewater via their stools or other body fluids [6, 7].

In this study, 2 postgrit primary influent samples were collected weekly starting June 2022—with reporting of results to TPH starting in October 2022—from each of Toronto's 4 wastewater treatment plants and sent to the National Microbiology Laboratory (NML) in Winnipeg, Canada, for analysis. In brief, the pellet mass harvested by centrifugation from approximately 120 mL of wastewater was processed via the MagAttract PowerMicrobiome DNA/RNA extraction kit (Qiagen) according to the manufacturer's instructions to yield a nucleic acid extract. Subsequent real-time polymerase chain reaction (PCR)–based quantification of 3 independent mpox assays targeting the tumor necrosis factor receptor gene terminal repeats was performed in triplicate: the G2R_G and G2R_WA assays developed by the US Centers for Disease Control and Prevention (CDC) and the G2R_NML assay developed by the NML. See Mejia et al [8] for the processing method.

### WGS and Phylogenetic Analysis

WGS of pathogens has been used in public health for outbreak investigations and surveillance [9]. Genomic epidemiology uses pathogen genomics to infer transmission chains and can bring more precision to public health investigations.

In this study, clinical specimens were received as part of clinical diagnostic services and surveillance programs at Public Health Ontario. MPXV real-time PCR was performed as previously described on the following clinical specimens: skin or mucosal lesion and throat/oral, nasopharyngeal, and genital (anal, penile, perineal, rectal, or scrotal) swabs [10]. Clinical specimens from October 2022 to June 2023 were all sequenced if real-time PCR cycle threshold values were <30 and sufficient volume remained.

Specimens from June to September 2022 were selected as part of a convenience sampling strategy. We used a targeted amplification–based WGS of MPXV directly on clinical specimens (98 primers producing tiled 5-kb overlapping amplicons). The normalized libraries were prepared with the Nextera XT DNA Library Preparation Kit (Illumina Inc) and sequenced on Illumina MiniSeq as previously described [11]. We downloaded sequences from GISAID (https://gisaid.org/) with percentage identity >99.5% for all the sequences from Toronto (25 July 2023). In addition, all 223 MPXV sequences on GISAID in the lineage B.1.2 were downloaded (16 February 2023), and we kept the 10 top hits. A maximum likelihood tree was generated with IQ-TREE (version 1.6.2) [12] with ModelFinder to select the best substitution model [12, 13]. Statistical branch support was inferred by 10 000 ultrafast replicates.

### Comparative Epidemic Appraisal

Surveillance data up to 15 December 2022 were used to describe the initial outbreak, and subsequent cases and data were used to describe the 2022–2023 resurgence.

### Ethics Statement

This activity was determined by TPH to meet criteria for public health surveillance and evaluation related to managing the outbreak; therefore, no institutional review board approval was required, and informed consent was not applicable.

## RESULTS

### Case-Based Surveillance

**Figure 1.**
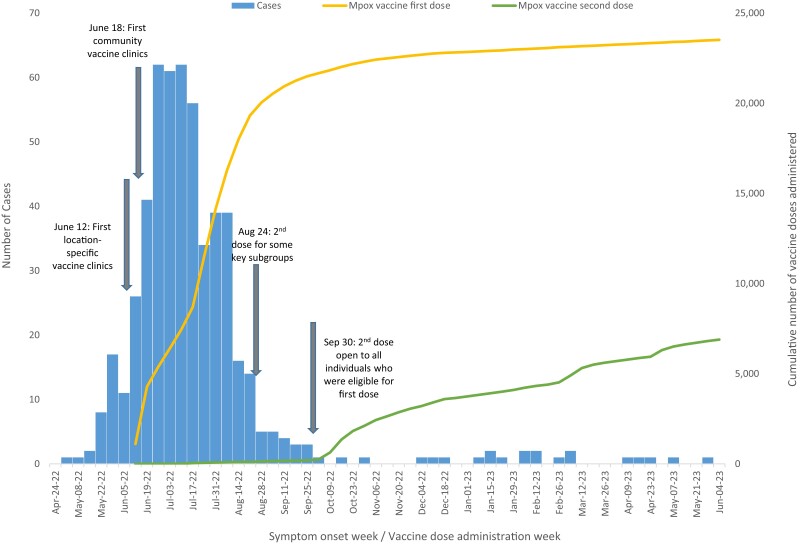
Epidemic curve of reported mpox cases in Toronto residents with an overlay of residents receiving vaccine doses administered in the city.

As of 19 June 2023 there were 515 mpox cases (507 confirmed, 8 probable) reported in the initial 2022 outbreak in Toronto and 17 cases in the resurgence that started in December and went into 2023 (Figure 1 and Supplementary Table).

The majority of cases were male: 99% in the initial outbreak and 100% in the resurgence. The median age was slightly younger in the initial outbreak (35 years; range, 17–74) as compared with the resurgence (40 years; range, 21–70). Of the male cases with sufficient information, 406 of 415 (98%) in the initial outbreak reported sex with men. All cases in the resurgence were gbMSM. In the initial outbreak and the resurgence, the most frequently reported risk factor after sex with the same sex was >1 sex contact in the last 6 months (61%). There was no evidence of transmission among household contacts.

Thirteen cases were hospitalized, all during the initial outbreak. The median length of hospital stay was 5 days (range, <1–14). No deaths were reported.

### Vaccine Administration

Vaccines were administered in public health clinics and by community health care providers. First and second doses were administered subcutaneously. A total of 26 487 doses of mpox vaccine were administered to Toronto residents in 2022 and 4185 in the first half of 2023; 77% were first doses and 23% were second.

A campaign was instituted to increase second-dose vaccine completion rates. A media release was issued at the time of the resurgence, and direct reminders were sent to recipients of a single vaccine dose who were overdue for a second. The latter strategy was repeated leading up to Toronto Pride festivities in June 2023. The proportion of fully vaccinated individuals out of those initiating vaccination increased from 16% to >30% from the detection of the resurgence to 30 June 2023.

### Wastewater-Based Surveillance

**Figure 2.**
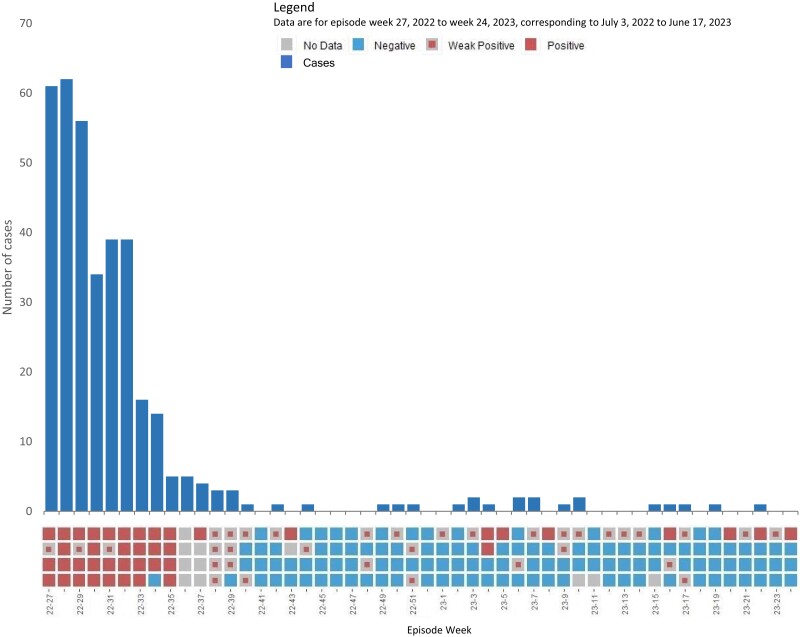
Mpox wastewater positivity matrix by collection site (4 sites). Laboratory data are aggregated to the epiweek. The epiweek was regarded as negative if there were no detections across the 3 assays employed in this work (G2R_G, G2R_WA, G2R_NML) for all samples tested that week, where each assay is tested in technical triplicate (9 replicates for each sample). The epiweek was regarded as a weak positive if the average replicate positivity across all assays and samples was ≤11%. The epiweek was regarded as positive if the average replicate positivity was >11% across all assays and samples. If no samples were collected in the corresponding period, the week is regarded as no data.

For the duration of reporting wastewater results to TPH, there was a temporal correlation between case reporting and positive detections across sample sites (Figure 2). During the initial outbreak period, mpox was robustly detected throughout the city across all wastewater catchments. This period corresponded to the highest case load (epiweeks 22–35). Cases subsequently fell, and the detection of MPXV in wastewater exhibited a largely weak positive detection; in the period between the initial outbreak and the resurgence (epiweeks 45–47), all wastewater sampling results were negative, corresponding to a period where no cases were reported. During the period of resurgence, consistent weak and positive detections were detected.

### Whole Genome Sequencing

In the spring/summer outbreak of 2022 and the 2022–2023 resurgence, WGS was performed on 88 of 505 (17.4%) and 14 of 17 (82.4%) laboratory-confirmed mpox cases from Toronto, respectively. We identified 7 MPXV lineages: B.1, B.1.1, B.1.2, B.1.4, B.1.7, B.1.8, and B.1.12. Starting in December 2022, MPXV lineage B.1.2 became predominant in Toronto with a total of 12 cases, which had not been the case before (Supplementary Figure 1). MPXV lineage B.1.2 had not been found in the sequences from Ontario since August 2022, and these 2 cases were not genetically related to the new cluster. Specifically, the B.1.2 cases from the December–January cluster, with 2 sequences from another country, were further apart from the only other 2 prior mpox cases of the same lineage from Ontario (PHOL131 and PHOL053; collected in August 2022). No epilinks between resurgent cases and the genomically linked country were identified. Ongoing cases of MPXV B.1.2 were detected until May 2023.

## DISCUSSION

Based on the epidemiologic data collected, the initial outbreak and subsequent small resurgence affected gbMSM as a result of propagated close contact transmission. The WGS results suggest propagated transmission of distinct lineages in the initial outbreak and resurgence. The WGS findings suggest new importations as the source for the resurgent cases. Between the initial outbreak and resurgence, there was a temporal association in the absence of clinical case reporting and the absent detection of wastewater MPXV in fall 2022. Based on the WGS lineages pointing to importation and 2 distinct clusters, as well as the absence of positive WBS results preceding the second outbreak, it is unlikely that undetected local transmission led to the resurgence of new case reports in December 2022.

At least one other study has speculated importation as a factor in local mpox resurgence based on the history of a known epilink [14]. Genomic sequencing results, if available, can be helpful in validating such transmission hypotheses. When facing novel pathogens, public health officials must address several important disease control questions. Mpox had not circulated globally prior to spring 2022, and critical questions included the amount of underreporting and underdetection occurring in our community. Traditional surveillance systems based on notification of clinical cases fail to capture cases at 2 distinct levels: from the community, since not all cases are symptomatic and not all symptomatic individuals seek health care, and at the health care level, due to inadequate diagnosis and/or reporting of cases among patients who sought medical advice [15]. Mpox is vulnerable to both these issues. The disease can present as self-limited and similar to other sexually transmitted infections; stigma and fear of reporting may lead to patients not seeking clinical care. Isolation requirements for mpox per local case management guidelines can further give people pause when seeking care. People with mpox may not identify with the at-risk group, and case patients and health care providers may not include the disease on their differential. In addition, as the infection was emerging locally, it was unclear whether asymptomatic MPXV infections were occurring. A recent study from the Netherlands used clinical data and WGS to consider whether its outbreak may have started earlier than detected, and the authors concluded that undetected local transmission was unlikely prior to May 2022 [16].

There are many examples where WGS of different pathogens affected public health decision making [17]. More recently, identical MPXV WGS confirmed transmission between a patient and a health care worker, which led to novel mpox-specific infection control measures [18]. In Canada, wastewater has been used for surveillance of SARS-CoV-2, particularly once clinical testing reduced dramatically [6].

From our vaccine surveillance data and monitoring, it was clear that further work could be done to improve second-dose vaccine completion. Following implementation of a second-dose campaign, second-dose completion of those who initiated first doses rose to 30%. In our study, we report on vaccine completion rates for those who initiated immunization rather than vaccine coverage. Our estimated vaccine coverage was based on a midpoint and range of denominators drawn from prior research and population surveys [19, 20] for Canada, which may not be an accurate reflection of Toronto's sexually active gbMSM population at the time of this outbreak. By early summer 2023, we estimate that our first-dose coverage was 48.7% (range of 40.7%–60.5%) and our second dose 14.6% (range of 12.2%–18.1%), but these estimates are challenged by the lack of an up-to-date and accurate denominator specific to Toronto. Vaccine coverage is an important indicator, as it may affect the risk and size of future outbreaks. A CDC analysis demonstrating the relationship between population mpox immunity and the risk for outbreak recurrence underscored the need for sustained mpox vaccination services, particularly in communities with low vaccination coverage [21]. Recent CDC vaccine coverage estimates demonstrated a range of state-wide coverage, from 4.6% to 66.2%, with 23% of the at-risk population estimated to be fully vaccinated in the United States [22].

In our jurisdiction of Toronto, there was a long lag (about 3 months) between the initial first-dose campaign and second-dose eligibility. This, coupled with declining case activity and less media attention, may have contributed to complacency and confusion about second-dose eligibility and poorer second-dose uptake.

As described elsewhere, behavioral changes within the community at risk were likely a major factor in controlling the initial outbreak in Toronto [23]. Recent modeling studies examining the effects of control measures on transmission dynamics found that infection-induced immunity and behavioral changes among gbMSM were the primary reasons why outbreaks faded in nonendemic regions, with vaccination playing an important role in the risk of minimizing future resurgence [24, 25]. Given the low vaccine coverage and new susceptible persons entering transmission networks, resurgences will possibly occur in prior nonendemic areas, and enhanced surveillance, community engagement, risk communication, and vaccine promotion have an ongoing role.

A strength of conducting epidemic appraisals such as this is that there are limitations to single-system platforms: clinical surveillance, WGS, and WBS each have strengths and limitations. Combining information from these different sources was needed to better understand the epidemiology and evolution of transmission in a previously nonendemic area. As such, a way forward for public health in responding to zoonotic pathogens with the potential for endemicity in new species in previously nonendemic areas is to consider the benefits of combining forms of surveillance and to further validate these methods through analysis and research. WGS and WBS systems can be very resource intensive; therefore, it is essential to continue to study their applications for timely public health action.

### Limitations

Our investigation is subject to a number of limitations. Vaccine administration data are available only for doses administered in Toronto. An unpublished analysis showed that the number of Toronto residents who received vaccine within Ontario but outside Toronto represented just 0.7% of all doses; therefore, it is not anticipated that these doses would significantly affect our results. Data for vaccine doses administered outside Ontario are not available. If a significant proportion of our population received second doses outside Ontario, then our proportion of fully vaccinated individuals out of those initiating vaccine would be an underestimate. Data collection of social determinants of health, such as race or income, was not done during vaccine administration, so disparities in such coverage cannot be reported.

WGS was performed on only 19.2% of the mpox cases. The proportion of cases sequenced increased as the case count decreased and, during the 2022–2023 resurgence, represented 82.4% of reported cases. The multiple lineages identified and their temporality suggest ongoing importation of mpox within the region. The number of publicly available sequences worldwide represent about 8.5% (7530/88 600) of cases confirmed by the World Health Organization and limit our interpretation of importation [26].

Wastewater surveillance for MPXV [27] has emerged from the significant global effort to monitor SARS-CoV-2 according to this method, where studies of viral shedding kinetics and stability and multiyear surveillance efforts have made possible the development of forecasting models. Similar studies for the application of WBS to mpox control are lacking; this makes interpretation and specifically the estimation of the disparity between wastewater signals and clinical cases difficult. As reported by other groups, the inherently low signal intensity observed in mpox-positive catchments makes a quantitative-based interpretation of the signal difficult [28]. Encouraging findings in the work presented here include the persistent wastewater-based detections while the case burden was low during the resurgence period, especially when considering that Toronto represents the largest wastewater catchment in Canada and is prone to dilution of signals given its large population. Moderate improvements in the laboratory detection of mpox could yield improved detection sufficient to better monitor and provide advance detection of future waves of community outbreaks. Further work is needed to understand the etiology of weak persistent signals in catchments.

As mentioned in the discussion, clinical detection of cases is subject to limitations at the patient and health care provider levels. Mpox case management was done separately from HIV/sexually transmitted infection case management, and analysis of coinfections and interventions for coinfections were not done for the purposes of this study. Only cases of Toronto residents would be reported to TPH; however, supplementing surveillance with highly sensitive WBS and WGS may allow better understanding of importations of disease, transmission dynamics, and the frequency and scale of locally unreported transmission.

## Supplementary Data

Supplementary materials are available at *The Journal of Infectious Diseases* online (http://jid.oxfordjournals.org/). Supplementary materials consist of data provided by the author that are published to benefit the reader. The posted materials are not copyedited. The contents of all supplementary data are the sole responsibility of the authors. Questions or messages regarding errors should be addressed to the author.

## Supplementary Material

jiad533_Supplementary_Data

## Notes

***Acknowledgments.*** TPH mpox outbreak control team including the following staff for their help with this manuscript: Les Shulman, Victoria Mielke, Karem Batista, Donna Ansara, Jasmine Ho, Mary Ann Gatbonton; the Gay Men's Sexual Health Alliance, Ontario; and the Ministry of Health, Ontario. Codey Dueck (Wastewater Surveillance Unit, Public Health Agency of Canada) for assistance in the preparation of figures. The city of Toronto for providing wastewater samples for analysis. We thank Audra Nagasawa and Statistics Canada's Canadian Wastewater Survey for assistance with sample and data collection. From Public Health Ontario and Public Health Ontario Laboratory, we thank Venkata R. Duvvuri, Kirby Cronin, Samir N. Patel, and Jessica P. Hopkins for their contributions to the genomics sections. The institutions of H. S., S. I., and C. S. M. carry out the surveillance activities that produce the data used for this work. D. H. S. T. holds a Tier 2 Canada Research Chair in HIV Prevention and STIs.

***Author contributions.*** H. S. and R. Shahin designed the study, and H. S. led the manuscript development. H. S., R. Shahin, S. O., S. I., C. S. M., R. Stuart, S. P., A. C., S. M., D. H. S. T, and M. F. reviewed drafts of the manuscript for important intellectual content and finalized the manuscript. S. O. led the case and vaccine descriptive analyses and results. S. I. led the genomics analyses and results. T. W. B. performed the genomics analyses and generated the genomics tree. A. E. developed the genomics sequencing method and performed genomics sequencing. C. S. M. led the wastewater analyses and results. E. M. M. developed the wastewater laboratory analysis. N. A. H. led the wastewater validation analysis.

***Financial support.*** No financial support was received for this work.

***Supplement sponsorship.*** This article appears as part of the supplement “Mpox: Challenges and Opportunities Following the Global 2022 Outbreak,” sponsored by the Centers for Disease Control and Prevention (Atlanta, GA).
