# Supplementary material for: Preparing for Mpox Resurgence: Surveillance Lessons From Outbreaks in Toronto, Canada
Source: J Infect Dis. 2023 Nov 30;229(Suppl 2):S305–12. doi: 10.1093/infdis/jiad533 (PMC10965211; doi:10.1093/infdis/jiad533)
Supplement: jiad533_Supplementary_Data [file jiad533_supplementary_data.zip › Supplementary_Table.docx]

**Supplementary Table**: Case Demographics and Case Characteristics by Mpox Outbreak Wave

|  | **Initial Outbreak** | **Resurgence** |
| --- | --- | --- |
| **Number of cases** | 515  508 lab-confirmed  8 probable | 17  17 lab-confirmed |
| **Case demographics** |  |  |
| Median age in years (range) | 35 (17-74) | 40 (21-70) |
| Male (%) | 99 | 100 |
| **Severity** |  |  |
| Number (%) hospital admissions | 13 (2.5) | 0 (0) |
| Number (%) intensive care unit admissions | 1 (0.2) | 0 (0) |
| Number of deaths | 0 | 0 |
| **Risk Factors*** |  |  |
| MSM among male cases (%) | 98 | 100 |
| Reporting more than one sex contact in last six months (%) | 61 | 81 |
| Reporting new contact in the past two months (%) | 56 | 81 |
| Reporting anonymous sex (%) | 53 | 81 |
| **Vaccination status**** |  |  |
| Partially vaccinated (one dose) (%) | 9 | 41 |
| Fully vaccinated (two doses) (%) | <1 | 18 |
| Unvaccinated (no doses) (%) | 91 | 41 |

*Only includes cases where sufficient data are available.

** Vaccination status at the time of illness onset was determined as fully vaccinated (two doses), partially vaccinated (one dose) or unvaccinated (no doses), adjusted by 14 days to account for the time required to mount an immune response.
