# Supplementary material for: Preparing for Mpox Resurgence: Surveillance Lessons From Outbreaks in Toronto, Canada
Source: J Infect Dis. 2023 Nov 30;229(Suppl 2):S305–12. doi: 10.1093/infdis/jiad533 (PMC10965211; doi:10.1093/infdis/jiad533)
Supplement: jiad533_Supplementary_Data [file jiad533_supplementary_data.zip › Supplementary_Figure_Description.docx]

**Supplementary Figure**: Maximum-likelihood tree of WGS of *Monkeypox virus* specimens from Toronto and references. A total of 102 sequences from Toronto and 18 references outside of Ontario were added with 195,833 positions included in the final dataset. The scale bar represents the number of nucleotide substitutions per site. Colours show specimens sequenced in this study belong to different MPXV lineages as specified. Phylogenetic trees were rooted using NCBI reference sequence MT903344.1.
