# Supplementary figures and images for: Preparing for Mpox Resurgence: Surveillance Lessons From Outbreaks in Toronto, Canada
Source: J Infect Dis. 2023 Nov 30;229(Suppl 2):S305–12. doi: 10.1093/infdis/jiad533 (PMC10965211; doi:10.1093/infdis/jiad533)

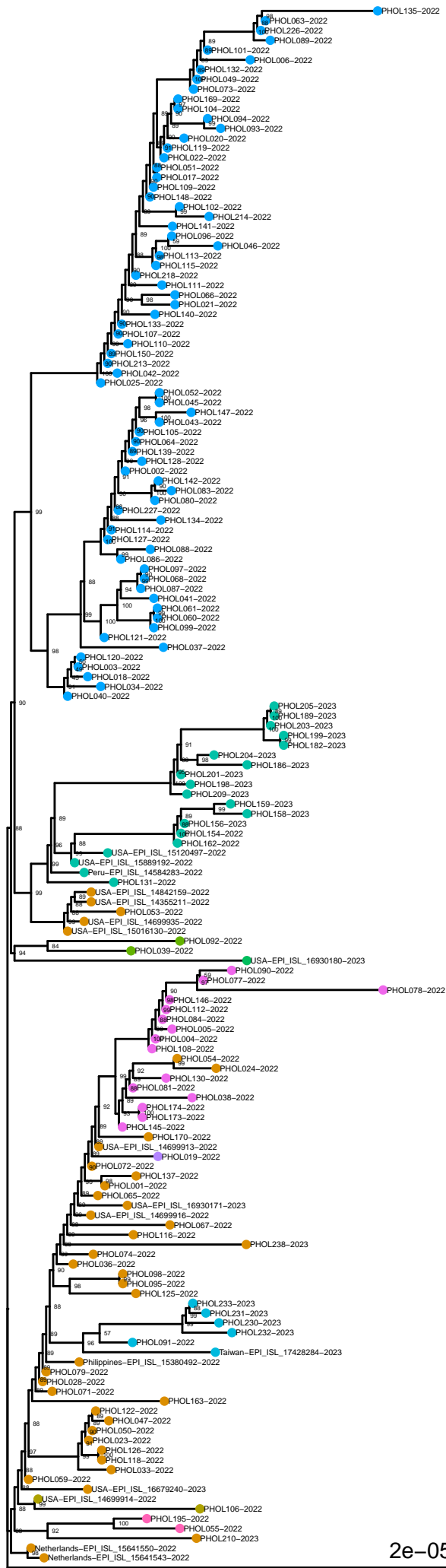

## Lineage

- A.1
- B.1
- B.1.1
- B.1.12
- B.1.17
- B.1.2
- B.1.3
- B.1.4
- B.1.5
- B.1.7
- B.1.8

Supplement: jiad533_Supplementary_Data [file jiad533_supplementary_data.zip › Supplementary_Figure.pdf]
